# Cross-talk between freezing response and signaling for regulatory transcriptions of *MIR475b* and its targets by miR475b promoter in *Populus suaveolens*

**DOI:** 10.1038/srep20648

**Published:** 2016-02-08

**Authors:** Jun Niu, Jia Wang, Huiwen Hu, Yinlei Chen, Jiyong An, Jian Cai, Runze Sun, Zhongting Sheng, Xieping Liu, Shanzhi Lin

**Affiliations:** 1 College of Biological Sciences and Biotechnology, National Engineering Laboratory for Tree Breeding, Key Laboratory of Genetics and Breeding in Forest Trees and Ornamental Plants, Ministry of Education, Beijing Forestry University, Beijing 10083, China

## Abstract

MicroRNAs (miRNAs) are small, non-coding RNAs that play important roles in post-transcriptional regulation of their target genes, yet the transcriptional regulation of plant miRNAs by promoter is poorly understood. Here, we firstly clone pri-miR475b cDNA and its native promoter from *P. suaveolens*, and characterize *Psu-MIR475b* as *class-II* gene transcribed by RNA polymerase II. By 5′ deletion analysis of Psu-miR475b promoter in a series of promoter-GUS chimeric vectors, we functionally identify three positive regulatory regions and multiple *cis*-acting elements responsible for Psu-miR475b promoter activity in response to freezing stress and exogenous hormone treatment. Moreover, the Psu-miR475b promoter activity displays a tissue-specific manner, negatively regulated by freezing stress and positively by MeJA, SA or GA treatment. Importantly, we comparatively analyze the time-course transcriptional profiles of Psu-miR475b and its targets in Psu-miR475b over-expression transgenic plants controlled by Psu-miR475b-specific promoter or CaMV 35S constitutive promoter, and explore the regulatory mechanism of Psu-miR475b promoter controlling transcriptional expressions of *Psu-MIR475b* and its targets in response to freezing stress and exogenous hormone treatment. Our results reveal that Psu-miR475b promoter-mediated transcriptions of *Psu-MIR475b* and its targets in response to freezing stress may be involved in a cross-talk between freezing response and stress signaling process.

Low temperature, especially freezing (<0 °C), is one of the major environmental stresses that seriously influence in the growth, development, distribution and productivity of plants^1^,^2^. Freezing tolerance and cold acclimation are highly complex process involved in physiological and metabolic modifications for cold response and a multiple gene expression network controlling plant tolerance to cold stress^1^,^2^,^3^,^4^,^5^,^6^,^7^,^8^,^9^. However, the regulatory networks of overall response of plants to low temperature stress still remains unclear. MicroRNAs (miRNAs) are a highly conserved class of endogenous single-stranded small non-coding RNAs that have been clearly shown to serve as negative regulators to modulate plant gene expression at post-transcriptional level by transcript cleavage or translational repression of target genes^10^,^11^,^12^. In recent years, the significant alterations in transcript levels of some miRNAs have been identified in response to cold stress in several plants such as Arabidopsis^5^,^13^,^14^,^15^, rice^16^, wheat^17^, *Setaria italica*^18^, *Phaseolus vulgaris*^19^, *Brachypodium distachyon*^20^, trifoliate orange^21^, celery^21^, *Camellia sinensis*^23^ and *Populus*^24^,^25^,^26^,^27^. Moreover, many predicted and experimentally confirmed targets of cold-responsive miRNAs encode a variety of transcription factors or other regulatory proteins implicated in low temperature response^20^,^21^,^22^,^24^,^26^,^28^. Also, genetic transformation of miRNAs and their targets has been recently demonstrated to alter cold stress tolerance capacity in plants^28^,^29^. All of these findings have shown a crucial role of miRNAs in the regulation of gene expression in response of plants to low temperature. Thus, an importance for understanding the initiation and regulation of miRNA gene transcription under low temperature stress.

The biogenesis of miRNAs is complex. Most plant miRNA genes (*MIR*), located in intergenic regions, are transcribed as independent transcriptional units by RNA polymerase II (Pol II) to produce primary transcripts (pri-miRNA) and then processed into stem-loop structured miRNA precursors (pre-miRNAs) by Dicer-like enzyme 1 (DCL1) in the nucleus^10^,^11^,^12^,^30^,^31^,^32^,^33^,^34^. The mature miRNAs, transported to the cytoplasm, are incorporated into the RNA-induced silencing complex (RISC), and then lead to post-transcriptional gene silencing via transcript cleavage or/and translational repression of their target mRNAs by recognizing the base-pairing and interaction with their cognate targets^10^,^11^,^12^,^35^,^36^,^37^,^38^,^39^,^40^,^41^,^42^. Although much effort has been focused on elucidating the regulatory function of plant miRNAs, little is known about how *MIR* genes themselves are regulated. Recently, some studies have shown that plant miRNAs have the class II promoters and may be regulated by a similar mechanism as established for protein-coding genes. The promoters of miRNAs have been predicted in rice by bioinformatic analysis and *A. thaliana* by 5′ RACE, respectively^31^,^32^,^43^,^44^,^45^, indicating the promoter as a crucial control region for the transcription initiation of miRNAs. However, direct evidence for transcriptional regulation of *MIR* genes by its native promoter is very little to date. Thus, the nature of miRNA promoter remains one of the most interesting open problems in the study of miRNA biogenesis.

*Populus suaveolens*, a typical freezing-resistant arbor tree of poplar species, can survive under a freezing temperature of approximately −43.5 °C in winter in the distribution of eastern Siberia regions and Great Xing’an Mountain, Northeast of China, and has emerged as a novel ideal model plant to study the freezing resistance mechanism in woody plants^25^,^46^,^47^. Previously, we identified miR475b with a significant down-regulation in *P. suaveolens* under freezing stress (°C), and revealed that miR475b plays an important role in freezing resistance of *P. suaveolens*^25^,^27^. In this continued study, we report the clone and analysis of miR475b gene and its native promoter from *P. suaveolens*, and explore the tissue-specific expression pattern of Psu-miR475b promoter. Also, we produce a series of 5′ promoter deletion-GUS reporter constructs, and perform a combination of the histochemical and fluorometric GUS assay and qRT-PCR to functionally characterize a set of regulatory regions and *cis*-acting elements responsible for the transcriptional activity of Psu-miR475b promoter. Importantly, we comparatively analyze the time-course transcriptional expression profiles of Psu-miR475b and its target genes in Psu-miR475b over-expression transgenic plants controlled by Psu-miR475b-specific promoter or cauliflower mosaic virus 35S (CaMV 35S) constitutive promoter, and investigate the regulatory mechanism of Psu-miR475b promoter for the transcripts of *Psu-MIR475b* and its targets in the transgenic plants subjected to freezing stress and exogenous hormone treatment. To our knowledge, this is the first report of functional identification and regulatory mechanism of Psu-miR475b promoter governing the transcriptional expressions of *Psu-MIR475b* and its targets in response to freezing stress.

## Results

### Cloning and analysis of freezing-responsive Psu-miR475b and its promoter

**Figure 1:**
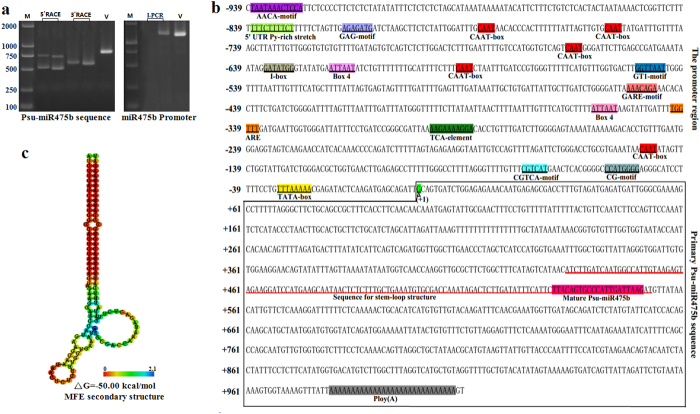
Cloning and analysis of freezing-responsive Psu-miR475b and its promoter from *P. suaveolens*. (**a**) Amplification of *Psu-MIR475b* gene (left) and its promoter (right) from *P. suaveolens* by 5′/3′RACE-PCR and I-PCR, respectively. M means the marker of 2000, and V means the validation for the full-length sequence of miR475b (left) and promoter (right). (**b**) Analysis of *Psu-MIR475b* gene sequence and *cis*-acting elements of promoter. The putative transcriptional start site (TSS) taken as +1 was marked with an upward filled triangle, and the regions from −939 to −1 relative to TSS was for Psu-miR475b promoter. The putative multiple *cis*-acting elements in Psu-miR475b promoter were underlined and presented with different background. The sequence of primary Psu-miR475b was located in the black box, where mature Psu-miR475b was marked with pink background, and the sequence of stem-loop structure was underlined with red. (**c**) Predicted stem-loop structure of Psu-miR475b precursor.

To elucidate the regulatory mechanism of miR475b transcription in response of *P. suaveolens* to freezing stress, the 1011-bp full-length freezing-responsive pri-miRNA475b with a putative 5′-cap structure and 3′-poly(A) tail (designated as *Psu-MIR475b*, Accession No. JX262380) was first cloned by 5′ and 3′ RACE from *P. suaveolens* cDNA (Fig. 1a,b). In order to gain insights into miR475b transcription, the secondary structure of RNA sequences generated from pri-miR475b cDNA was analyzed. We found that miR475b precursor has folding back free energy of −50.00 kcal/mol to form a stable stem-loop structure, and its mature sequence with 21nt length (5′-UUACAGTGCCCATTGATTAAG-3′) located in 3′ arm of stem-loop structure (Fig. 1c). Importantly, based on 5′ end sequence of *Psu-MIR475b* gene, we used inversion-PCR (IPCR) to obtain full-length (939bp) Psu-miR475b promoter (Accession No. KM288552) from *P. suaveolens* genomic DNA (Fig. 1a,b).

Promoter is a crucial control region for transcription initiation of miRNAs. To understand the mechanism of the activation of *Psu-MIR475b* gene, it is required to locate *cis-*acting elements within its promoter region. By online programs, we characterized one core promoter element TATA box-like sequence (TTTAAAAA, −32/−25), five CAAT-boxes as common *cis*-acting elements (−149/−146, −591/−588, −666/−663, −757/−754, −786/−783), five light responsive elements [CG motif (CCATGGGG, −57/−50), Box 4 (ATTAAT, −360/−355, −620/−615), GT1-motif (GGTTAAT, −550/−544), I-box (−635/−629) and GAG-motif (AGAGATG, −817/−811)], four stress-related elements [CGTCA-motif (CGTCA, −75/−71), TCA-element (AAGAAAAGGA, −297/−288), GARE-motif (AAACAGA, −452/−446) and TC-rich repeat (AAACAGA, −687/−678)], two *cis*-acting regulatory elements [5′UTR Py-rich stretch (TTTCTTTTCT, −838/−829) and ARE (TGGTTT, −342/−339)], and one endosperm-specific regulatory AACA-motif (AATCTAATTT, −590/−581) within Psu-miR475b promoter (Fig. 1b and Table S1). Thus, an enrichment of diverse regulatory *cis*-acting elements in Psu-miR475b promoter.

### Tissue-specific activity for Psu-miR475b promoter in plants

**Figure 2:**
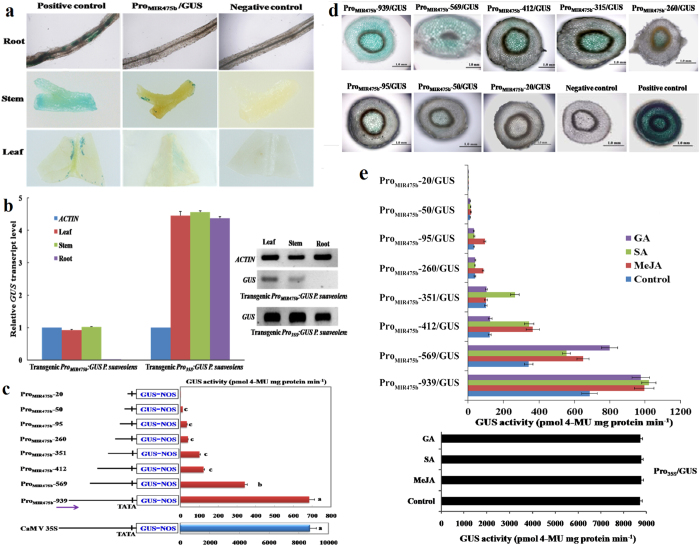
Characterizations of tissue specificity and multiple *cis*-regulatory elements for Psu-miR475b promoter. (**a**) Tissue-specific activity for Psu-miR475b promoter. The histochemical GUS staining in different tissues of transgenic tobacco shows that Psu-miR475b promoter similar to those of protein-coding genes is able to drive *GUS* expression but with a tissue-specific manner. GUS staining from CaMV 35S (pBI121 vector) transformant and wild-type tobacco were served as positive and negative controls, respectively. (**b**) The expression analysis of *GUS* reporter gene in different tissues of transgenic *P. suaveolens* plants by RT-PCR and qRT-PCR. The poplar *ACTIN* gene was used as an endogenous reference gene and its expression level was arbitrarily set to 1.00 for standardization. The means and standard deviations of the relative GUS transcript levels in the respective tissue are shown. (**c**) 5′ deletion analysis of Psu-miR475b promoter by the fluorometric GUS assay in transgenic tobacco stem. (**d**) 5′ deletion analysis of Psu-miR475b promoter by histochemical GUS staining in transgenic tobacco stem (as one predominant tissue). (**e**) GUS activity driven by the Psu-miR475b promoter in stem of transgenic tobacco plants subjected to MeJA, GA or SA. GUS activity from the CaMV 35S (pBI121 vector) transformants served as a comparison. Data are mean and standard deviations of twelve transgenic lines. The numbers below the bars indicate the fold changes of GUS activity. Significance of the changes produced after each treatment was assessed using Student’s t tests (*P < 0.05, **P < 0.01).

To explore whether Psu-miR475b promoter was similar to those of protein-coding genes, 939 bp full-length Psu-miR475b promoter (Pro_MIR475b_) and CaMV 35S promoter (Pro_35S_) were respectively fused to *GUS* reporter gene and transferred into tobacco. We compared GUS activity in different tissues of transgenic *Pro*_*MIR475b*_*:GUS* and *Pro*_*35S*_*:GUS* tobacco subjected to histochemical GUS staining. The *Pro*_*MIR475b*_*:GUS* plants exhibited *GUS* expression in the stems and leaves, but no GUS staining was detected in the roots (Fig. 2a). In stark contrast with *Pro*_*MIR475b*_*:GUS* plants, all tested tissues of *Pro*_*35S*_*:GUS* lines displayed a significant higher *GUS* expression (Fig. 2a). These results indicate that Psu-miR475b promoter is able to direct *GUS* gene expression, but differs from CaMV 35S constitutive promoter that served as positive control, directing a stronger expression of *GUS*.

Previously, we identified one preferential tissue-specific transcript of Psu-miR475b in the leaves and stems of *P. suaveolens*^27^. Thus, it is need to illustrate the tissue-specificity of Psu-miR475b promoter in *P. suaveolens* to address the regulatory mechanism of Psu-miR475b promoter controlling the transcription of Psu-miR475b. Here, the constructed promoter-GUS chimeric vectors (*Pro*_*MIR475b*_*:GUS* and *Pro*_*35S*_*:GUS*) were transferred into *P. suaveolens* for the transcript level assay of *GUS* gene in different tissues by RT-PCR and qRT-PCR (Fig. 2b). As expected, Psu-miR475b promoter-driving *GUS* gene was transcribed in the leaves and stems of transgenic *P. suaveolens*, but no transcript observed for the roots, which was comparable with those observations in transgenic *Pro*_*MIR475b*_*:GUS* tobacco (Fig. 2a). In addition, transgenic *P. suaveolens* driven by 35S promoter greatly increased GUS activity in all tissues examined (Fig. 2b). Thus, our findings reveal a typical tissue-specific expression pattern for Psu-miR475b promoter in plants.

### Characterization of multiple *cis*-regulatory elements in Psu-miR475b promoter

To understand the regulatory mechanism controlling *Psu-MIR475b* gene expression by its native promoter, we first sought to determine the functionality of our predicted regulatory regions responsible for the Psu-miR475b promoter activity. Hence, we produced a series of 5′ promoter deletion-GUS constructs, covering different regions from −939 to −1, −569 to −1, −412 to −1, −351 to −1, −260 to −1, −95 to −1, −50 to −1 and −20 to −1 (Figure S1). The multiple transgenic tobacco plants (>12 independent lines) were obtained (Figure S2), and their stems were used as one predominant tissues (Fig. 2a) for the *GUS* expression analysis. The fluorometric GUS assay clearly demonstrated that compared with full-length promoter (939 bp), the deletion from −939 (relative to TSS) to −570, −569 to −413, −351 to −261, and −95 to −51 caused a significant reduction (about 1.0-, 1.8-, 1.6-, and 1.8-fold, respectively) in GUS activity in transgenic tobacco stems, whereas only 0.2-fold decrease was observed for the deletion from −412 to −352, and −261 to −96 (Fig. 2c). Intriguingly, *GUS* expression from −50 to −1 was significantly lower than others, while further deletion to −20 abolished the *GUS* expression (Fig. 2c). These investigations were entirely consistent with our results of histochemical GUS staining in the stems of transgenic tobacco plants (Fig. 2d). Thus, our data indicate that three positive regulatory regions (−939 to −413, −351 to −261 and −95 to −51) are responsible for the basal activity of Psu-miR475b promoter, and one region (−50 to −21) required for transcriptional initiation.

As we were surprised by a cluster of stress-related *cis*-elements within Psu-miR475b promoter (Fig. 1b and Table S1), we attempted to establish whether or not the basal activity of Psu-miR475b promoter could be affected by the treatments of stress-related stimuli. To achieve this, a series of 5′ promoter deletion-GUS transgenic tobacco was subjected to the treatments of MeJA, SA, ABA and GA, respectively. By using fluorometric GUS assay, an obvious induction of GUS activity was observed in the stems of Pro_MIR475b_-939/GUS and Pro_MIR475b_-569/GUS plants upon treatment by SA, MeJA and GA, but a lower inducible ratio for Pro_MIR475b_-569/GUS plants. In contrast, the GA-inducible expression of *GUS* gene seemed to disappear in Pro_MIR475b_-412/GUS and Pro_MIR475b_-351/GUS lines, which responded to SA and MeJA treatments. Also, Pro_MIR475b_-260/GUS and Pro_MIR475b_-95/GUS plants exhibited a higher GUS activity in response to MeJA, but not GA and SA. Notably, no inducible expression was shown for Pro_MIR475b_-50/GUS and Pro_MIR475b_-20/GUS plants (Fig. 2e). The present results indicate that the regions from −569 to −413, −351 to −261, and −95 to −51 are respectively required for GA-, SA-, and MeJA-inducible activity of Psu-miR475b promoter. However, no response of GUS activity was observed with or without ABA treatment. It is also interesting to note that Pro_35S_/GUS lines showed no significant inducible expression under these imposed conditions (Fig. 2e).

### Cross-talk between freezing response and stress signaling for Pro_MIR475b_ activity regulation

**Figure 3:**
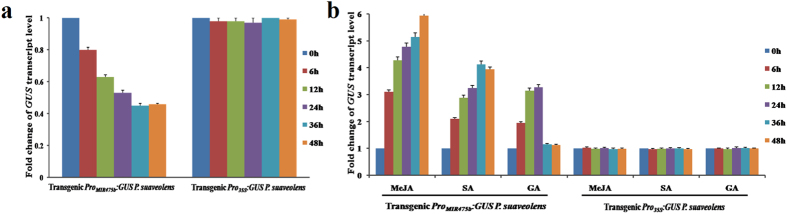
The impacts of freezing stress and defense-related stimuli treatments on the activity of Psu-miR475b promoter in transgenic *Pro*_*MIR475b*_:*GUS* and *Pro*_*35S*_*:GUS P. suaveolens*. (**a**) The Psu-miR475b promoter activity is negatively regulated by freezing stress. (**b**) The Psu-miR475b promoter activity is induced by the applications of exogenous SA, MeJA and GA.

Recently, we identified the significant down-regulation of Psu-miR475b in response of *P. suaveolens* to 0 °C stress^25^, which allowed us to explore whether the activity of freezing-responsive Psu-miR475b promoter was specifically regulated by low temperature. Thus, we generated *Pro*_*MIR475b*_*:GUS* and *Pro*_*35S*_*:GUS* transgenic *P. suaveolens* (Figure S2), and subjected them to the time-course analysis of *GUS* transcript level by qRT-PCR after 0 °C treatment for 0–48 h. We observed that during freezing-stress treatment, Pro_MIR475b_-driving *GUS* expression was significantly decreased from 6 to 48 h, while no expression change was directed by CaMV 35S promoter in transgenic plants (Fig. 3a), indicating that Psu-miR475b promoter activity was specifically down-regulated by freezing stress. In addition, to investigate whether the signaling pathway was involved in the activity regulation of Psu-miR475b promoter, our analysis focuses on identifying the regulatory patterns of Psu-miR475b promoter by MeJA, SA and GA. In the case of treatment with exogenous MeJA, SA or GA for 0–48 h, we found that that in *Pro*_*MIR475b*_*:GUS* plants, *GUS* expression after MeJA treatment was more sustained and continued to increase over the time points tested, while the relative low transcript of GUS with a peak value at 36 h was detected for SA treatment. By contrast, when exposed to GA, *GUS* expression first slightly increased and then returned to a normal level after 24 h (Fig. 3b). However, *Pro*_*35S*_*:GUS* plants showed no inducible expression of *GUS* by all of the imposed conditions (Fig. 3). These results indicate that Psu-miR475b promoter activity can be induced by SA, MeJA and GA, but with differential time-course regulatory manner respond to different hormones.

The above investigations prompt us to explore whether freezing response was linked to the hormone signal. Here, we also examine *GUS* expression driven by Psu-miR475b promoter with serial 5′ deletions under freezing stress for 48 h. Notably, all deletion constructs except Pro_MIR475b_-20 and Pro_MIR475b_-50 showed an obvious decrease of GUS activity when treated with freezing, but no significant difference of *GUS* expression was observed between Pro_MIR475b_-95 and Pro_MIR475b_-260 or between Pro_MIR475b_-351 and Pro_MIR475b_-412 lines (Figure S3), indicating that the regions from −939 to −413, −351 to −261, and −95 to −51 are required for the regulation of Psu-miR475b promoter activity in response to freezing stress. Impressively, in those regions, MeJA-responsive CGTCA motif (−75/−71), SA-responsive TCA element (−297/−288) and GA-responsive GARE motif (−452/−446) were identified (Fig. 2e). Also, the Psu-miR475b promoter activity was induced by the GA, SA, and MeJA treatments (Fig. 3b). Thus, it could be concluded that a cross-talk between freezing-stress response and hormone signaling may involve in the activity regulation of Psu-miR475b promoter.

### Pro_MIR475b_-mediated transcriptions of Psu-miR475b and its targets in transgenic *P. suaveolen*s

**Figure 4:**
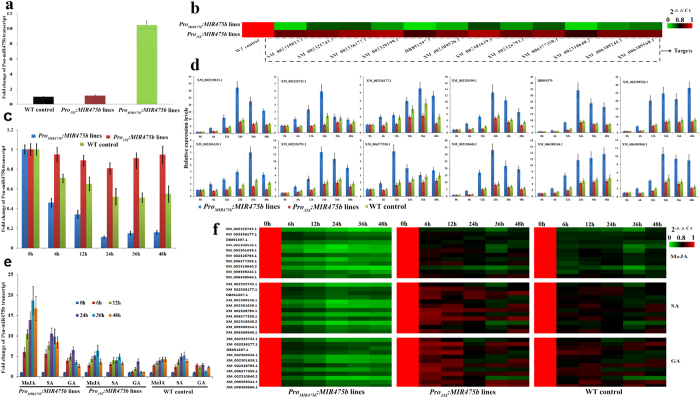
Transcriptional expression analysis of Psu-miR475b and its 12 targets in Psu-miR475b overexpression *P. suaveolens* by qRT-PCR. (**a**) Comparative analysis of Psu-miR475b transcription in transgenic *Pro*_*MIR475b*_*:MIR475b* and *Pro*_*35S*_*:MIR475b* plants, revealing an important contribution of Psu-miR475b promoter on the up-regulation of its native gene (*Psu-MIR475b*) transcription. Both miR167e and miR168a-3p were used as inner references. Error bars indicate standard deviations of three technical replicates. (**b**) Comparative analysis of miR475b-targeted genes in transgenic *Pro*_*MIR475b*_*:MIR475b* and *Pro*_*35S*_*:MIR475b* plants. The down-regulated expressions of all targets indicate that miR475b-targeted transcripts may be cleaved directly by Psu-miR475b. *ACTIN* gene was used as reference genes. The relative expression values in heatmap were counted as 2^−△△C t^, and the wild-type lines were used as the control. (**c**) The significantly down-regulated transcription of Psu-miR475b by freezing stress. (**d**) The significantly up-regulated transcription of all 12 targets by freezing stress. (**e**) The significantly induced transcription of Psu-miR475b by the MeJA, SA and GA treatments, which is tightly correlated with the stronger response of Psu-miR475b promoter to MeJA, SA and GA. (**f**) The significantly down-regulated transcription of all 12 targets by the MeJA, SA and GA treatments. The finding indicates an important regulatory role of Psu-miR475b promoter for Psu-miR475b-mediated transcriptional repression of its targets responsive to MeJA, SA and GA.

To address the regulatory mechanism of Psu-miR475b-specific promoter controlling *Psu-MIR475b* expression, we analyzed the time-course transcription pattern of Psu-miR475b by qRT-PCR in Psu-miR475b-overexpressing *P. suaveolens* under the control of Psu-miR475b promoter or CaMV 35S promoter (Figure S4). Compared with the wild-type (WT) controls, the most striking difference between two types of transgenic lines was observed in *Pro*_*MIR475b*_*:MIR475b* plants with about 6.1-fold increase of Psu-miR475b transcript, which is considerably higher than that (0.3-fold) in *Pro*_*35S*_*:MIR475b* lines (Fig. 4a), confirming an important contribution of Psu-miR475b promoter to regulate its native gene (*Psu-MIR475b*) transcription. Recently, we experimentally characterized 12 putative pentatricopeptide repeat protein (PPR) genes (XM_002319013.1, XM_002325743.1, XM_002336177.1, XM_002329199.1, DB891579, XM_002309526.1, XM_002301639.1, XM_002326793.1, XM_006377350.1, XM_002310640.2,XM_006389244.1, XM_006389560.1) as the targets of Psu-miR475b^27^. To understand how Psu-miR475b regulates the expressions of its targets, and to examine whether Psu-miR475b promoter was involved specifically in the regulation of miR475b-meidiated expression, we further checked the transcript levels of 12 miR475b-targeted *PPR* genes in transgenic plants. Compared to the WT controls, the transcripts of 12 *PPR* genes were all markedly reduced (4–9 fold) in *Pro*_*MIR475b*_*:MIR475b* plants, but no significant alteration was observed for *Pro*_*35S*_*:MIR475b* lines (Fig. 4b). This indicates that the expressions of miR475b-targeted genes in Psu-miR475b-overexpressing plants are specifically regulated by Psu-miR475b promoter. Also, the fact of an inverse correlation between Psu-miR475b (up-regulation) and its targets (down-regulation) (Fig. 4a,b) in transgenic plants showed that the miR475b-targeted transcripts may be cleaved directly by Psu-miR475b, which was confirmed by our previous 5′ RLM- RACE^27^.

### Pro_MIR475b_-mediated transcriptional regulation involved in freezing response and hormone signaling

Considering the fact that freezing stress has shown to confer a negative effect on the Psu-miR475b promoter-directed *GUS* expression (Fig. 3a), the obtained Psu-miR475b-overexpressing *P. suaveolens* were also exposed to 0 °C treatment for 0–48 h, and the temporal transcript profiles of Psu-miR475b and its targets were analyzed to reveal the regulatory mechanism of Psu-miR475b promoter for the transcriptional expressions of *Psu-MIR475b* and its targets in response to freezing stress. We found that the transcript level of Psu-miR475b in *Pro*_*MIR475b*_*:MIR475b* plants was lower after 6 h and greatly decreased with longer stress, while a small decline was detected in *Pro*_*35S*_*:MIR475b* lines with minimum valve at 24 h, similar to that of the WT controls (Fig. 4c). This finding reveals that Psu-miR475b transcription is altered in response to freezing stress, and a significant down-regulation is specifically driven by its native promoter. Also, the strongly induced expression of all 12 miR475b-targeted genes by freezing was identified in *Pro*_*MIR475b*_*:MIR475b* plants than in both *Pro*_*35S*_*:MIR475b* plants and WT controls (Fig. 4d), implying that the significant up-regulation of miR475b-targeted genes driven by Psu-miR475b promoter may be involved in freezing-stress response process.

Given that Psu-miR475b promoter activity is induced by MeJA, SA and GA (Fig. 3b), we attempted to address whether the application of these stimuli could trigger the transcriptions of Psu-miR475b and its targets. To this end, the transgenic *P. suaveolens* of *Pro*_*MIR475b*_*:MIR475b* and *Pro*_*35S*_*:MIR475b* were respectively treated by MeJA, SA or GA for 0–48 h. Here, Psu-miR475b transcript was up-regulated approximately 17.3-, 9.6- and 5.1-fold in *Pro*_*MIR475b*_*:MIR475b* plants by MeJA, SA and GA treatments for 48 h, peaked at 36, 24 and 24 h respectively, whereas a small induced expression for *Psu-MIR475b* was detected in both *Pro*_*35S*_*:MIR475b* lines and WT controls by these treatments (Fig. 4e). These results indicate that an obvious inducible transcription of Psu-miR475b by MeJA, SA and GA is tightly correlated with a stronger response of Psu-miR475b promoter to MeJA, SA and GA. It is also worth noticing that exogenous application of MeJA, SA or GA resulted in a nearly antiparallel transcript pattern of Psu-miR475b (up-regulation) and its targets (down-regulation) in transgenic *Pro*_*MIR475b*_*:MIR475b* plants, but the expressions of all targets remain stable in *Pro*_*35S*_*:MIR475b* lines under these imposed conditions (Fig. 4e,f).

## Discussion

In recent years, bioinformatic analysis has been applied to predict the promoters of miRNAs in Arabidopsis and rice^15^,^31^,^32^,^43^,^44^,^45^, but the experimental cloning and functional identification of *MIR* promoter was only reported for miR171a, miR172a and miR390a/b in Arabidopsis to data^48^,^49^,^50^. This study presents for the first time a regulatory mechanism of Psu-miR475b promoter governing the transcription of Psu-miR475b and its targets in response of *P. suaveolens* to freezing stress, where pri-miR475b cDNA and its promoter were firstly cloned from *P. suaveolens* (Fig. 1a,b). A lower folding back free energy was predicted for Psu-miR475b precursor (Fig. 1c), as reported in *Triticum aestivum*^51^, *B. distachyon*^20^ and *P. tomentosa*^26^. Intriguingly, the primary transcript of Psu-miR475b was capped at the 5′end and polyadenylated at the 3′ end (Fig. 1b), similar to the unique properties of *class-II* gene transcripts, which has been characterized in *A. thaliana MIR* genes^31^,^43^,^45^. Moreover, Psu-miR475b has one 21 nt-length mature sequence located in 3′ arm of stem-loop structure, and has uridine (U) as first nucleotide at 5′ end (Fig. 1b,c), which is entirely consistent with *P. trichocarpa* miR475a/b/c^52^, also identified as one characteristic feature of miRNAs in plants^20^,^24^,^31^. Together, all our findings reveal that *Psu-MIR475b* gene is transcribed as a single transcript unit by the RNA pol II mechanism.

The promoter contain essential components for the transcription regulation of *MIR* gene^31^,^32^. TATA-box, as a well-known core motif in the promoters of eukaryotic *class-II* genes, has been identified in most miRNA promoters of *A. thaliana* and *O. sativa*^31^,^32^,^43^,^44^,^45^, suggesting that most of plant *MIR* genes may present the same promoters as the protein-coding genes transcribed by RNA pol II. In this study, our identified one 8-nt TATA box-like sequence of Psu-miR475b promoter within −32 to −25 (Fig. 1c and Table S1) was compatible with those located in protein-coding genes, also correspond to authentic TATA box sequence within the core promoters of plant *MIR* genes^31^,^32^,^44^. Importantly, the up-regulated *GUS* was detected in both *Pro*_*MIR475b*_*:GUS* and *Pro*_*35S*_*:GUS* transgenic tobacco, but the relative lower GUS activity was observed for *Pro*_*MIR475b*_*:GUS* lines (Fig. 2c,d). All our results show indeed that Psu-miR475b promoter may be as pol II promoter, but exhibits a specificity for Psu-miR475b, which support the hypothesis of the differentiation between *MIR* genes and protein-coding genes^45^.

Earlier studies in four model species (*Caenorhabditis elegans, Homo sapiens, A. thaliana* and *O. sativa*) have revealed many significant conserved motifs in the promoters of *MIR* genes^32^. Recently, 11 over-represented *cis*-elements (AtMYC2, ARF, SORLREP3, G-box, SORLIP1, RY-repeat, LTRE, Evening element, TELO-box, DRE-like and AtMYB2), and 9 under-represented *cis*-elements (GATA box, LFY motif, T-box, GCC-box, RAV1-B, Bellringer BS3, CArG, HSEs and CCA1) were identified in the promoters of Arabidopsis *MIR* genes by position weight matrices (PWM)^45^. In this work, we performed the PWM method to identify a total of 13 *cis*-acting elements in Psu-miR475b promoter (Fig. 1b and Table S1), among which 11 elements (Box 4, GT1-motif, CGTCA-motif, GAG-motif, CG-motif, TCA-motif, GARE-motif, 5′UTR Py-rich stretch, TC-rich repeat, ARE and AACA-motif) were not previously reported. Our data indicate that these putative *cis*-regulatory elements may be specific to Psu-miR475b promoter, probably owing to *MIR* gene promoter with specific *cis*-acting elements for governing unique transcription of miRNA^53^. In addition, we present the first 5′ promoter progressive deletion analysis to elucidate the functionality of these potential regulatory *cis*-elements for the Psu-miR475b promoter activity. The histochemical and fluorometric GUS assay (Fig. 2c,d), combined with our characterizations of *cis*-acting elements in Psu-miR475b promoter (Fig. 1b and Table S1), suggests that CGTCA motif (involved in MaJA responsiveness), GAR-motif (GA responsiveness), TCA-element (SA responsiveness), TC-rich repeat (stress responsiveness) and 5′UTR Py-rich stretch (conferring high transcription level) may play the key role in the activity regulation of Psu-miR475b promoter, and TATA-box (as core promoter element) function as initiator for initiation of Psu-miR475b transcription. Importantly, the regions from −569 to −413, −351 to −261, and −95 to −51 were respectively required for GA-, SA-, and MeJA-inducible activity of Psu-miR475b promoter (Fig. 2e), also noted in the promoters of some cold-responsive miRNAs (such as miR167/393/408) in *A. thaliana*^14^. However, no response of GUS activity to ABA-treatment in transgenic plants may be correlated with the lack of ABA-responsive ABRE motif in the Psu-miR475b promoter (Table S1), as reported in the promoters of some *A. thaliana* miRNAs^14^, implying that the activity regulation of Psu-miR475b promoter may be ABA-independent pathway. Together, our deletion analysis suggest a complex regulatory mechanism for the activity of Psu-miR475b promoter controlled by its internal *cis*-acting elements.

Previously, miR475b has been shown to be down-regulated in response of *P. suaveolens* to freezing stress^27^. Our findings on the significant down-regulation of *GUS* gene by freezing stress in transgenic *Pro*_*MIR475b*_*:GUS* plants (Fig. 3a) and the obvious induced expression by the exogenous SA, MeJA and GA (Fig. 3b) reveal that Psu-miR475b promoter is one low temperature-responsive promoter, and its activity is negatively regulated by freezing stress and positively by the applications of exogenous hormones. This also prompt us to consider that there exists a complex relationship between freezing stress and hormone response signal, and a potential great cross-talk among MeJA, SA and GA for the activity regulation of Psu-miR475b promoter.

Recently, high-throughput sequencing revealed that some cold-induced miRNAs displayed up-regulation in response of *T. aestivum* to GA, ABA and JA^54^, suggesting that these miRNAs may involve in an intricate association between the signaling pathways and abiotic stress responses. In this study, the significant down-regulation for Psu-miR475b and up-regulation for its targets were characterized in *Pro*_*MIR475b*_*:MIR475b* transgenic *P. suaveolens* during freezing stress, while a slight alternation was observed for *Pro*_*35S*_*:MIR475b* plants (Fig. 4c,d), demonstrating that the transcriptions of Psu-miR475b and its targets driven by its native promoter are specifically altered in response to freezing stress. These results, combined with the finding that the promoter activity of Psu-miR475b is negatively regulated by freezing stress, confirm an involvement of Psu-miR475b promoter in the transcription regulation of *Psu-MIR475b* and its targets under freezing stress.

Many miRNAs were differentially regulated by exogenous application of plant hormones such as JA, SA, GA and ABA^13^,^54^. In this work, compared with *Pro*_*35S*_*:MIR475b* plants, the significant differential expressions of Psu-miR475b (up-regulation) and its targets (down-regulation) were identified in transgenic *Pro*_*MIR475b*_*:MIR475b* plants by the MeJA, SA and GA treatments (Fig. 4e,f), indicating that the exogenous hormones (MeJA, SA and GA) could regulate the transcripts of Psu-miR475b and its targets, likely though mediating the Psu-miR475b promoter activity. This could be supported by the fact of a higher inducible activity of Psu-miR475b promoter by the treatments of MeJA, SA and GA (Fig. 3b). Thus, we conclude that Psu-miR475b promoter-triggered transcriptions of *Psu-MIR475b* and its targets may be involved in the complex signaling pathways, mediated by MeJA, SA and GA.

Taken together, our findings that freezing stress-responsive Psu-miR475b and its targets as well as the Psu-miR475b promoter activity are prone to being affected by the treatments of freezing stress and exogenous MeJA, SA or GA, reveal the existence of a great cross-talk between freezing response and stress signaling process. It was suggested that the PPRs may provide a signaling link between mitochondrial electron transport and regulation of stress and hormonal responses in *A. thaliana*^24^. Microarray analysis of transcript expression has shown that many genes involved in the biosynthesis and signaling of plant endogenous hormone (such as JA, SA and GA) are down-regulated by cold stress in *A. thaliana*^5^. The previously characterized 12 PPRs as the targets for psu-miR475b^27^, integrated with all our investigations, suggest that the lower activity of Psu-miR475b promoter could be expected mainly due to the smaller biosynthesis capacity of endogenous hormone (MeJA, SA and GA) caused by freezing stress. Thus, the transcriptional expressions of Psu-miR475b and its targets by its native promoter may be involved in a complex signaling pathway and freezing-stressed response. Our findings also evidenced that Psu-miR475b-specific promoter is important determinant for the transcriptional regulation for Psu-miR475b and its targets in response of *P. suaveolens* to freezing stress.

## Methods

### Plant materials and growth conditions

*P. suaveolens*, obtained from Great Xing’an Mountain, Northeast of China, was used as the source material for this study. The plants were propagated by cutting and raised in pots within a controlled environment chamber (photoperiod: 16/8 h light/dark, minimum illumination: 0.2 mM s^−1^ m^−2^, day temperature: 20–30 °C) at Beijing Forestry University. Tissue-cultured *P. suaveolens* plants were raised and synchronized on modified MS medium as our previously described^55^. The fully developed leaves harvested from the tissue-cultured plantlets were subjected to genetic transformation. Tissue-culture tobacco (*Nicotiana tabacum* cv. W38) plants were performed on modified MS medium, and the fully developed tobacco leaves were then used for genetic transformation experiments.

### Freezing stress treatment of *P.suaveolens* plantlets

The 2-month-old plantlets with identical growth status were exposed to 0 °C, and the leaves were then harvested at time points 0, 6, 12, 24, 36 and 48 h post-treatment as described previously^25^. All materials were collected from three individual plants and immediately frozen in liquid nitrogen and stored at −80 °C.

### Cloning of pri-miR475b and its promoter and transformation of *P. suaveolens*

Total RNA was isolated from the tested samples using SV Total RNA Isolation System (Promega, Madison, WI, USA). The synthesis of first strand cDNA, and the 3′- and 5′-RACE of pri-miR475b were performed according to SMARTer RACE cDNA kit (Clontech, USA) illustrate, and then the purified amplification products were sequenced to assemble the full-length sequence of pri-miR475b. 3′- and 5′-RACE outer/inner primer could be seen in Supplementary Table S2. The full-length cDNA sequence (pri-miR475b) was amplified from *P. suaveolens* genomic DNA by PCR using two gene-specific primers 5′-AGGTAGTCAAGCACCATCACAAA-3′ (forward prime) and 5′-AACCTACAGCATGACCTAGAGGC-3′ (reverse primer), and then cloned into pGM-T Vector, giving pT-MIR475b. The sequence of the amplified DNA fragment was verified by sequencing. A XbaI-SacI fragment from pT-MIR475b containing the *MIR475b* sequence was then subcloned into the *Xba* I and *Sac* I sites of vector pBI121 between the CaMV 35S promoter and the *NOS* 3′poly (A) signal to generate the *35S:MIR475b* construct (named as *Pro*_*35S*_*:MIR475b*).

For full-length Psu-miR475b promoter from *P. suaveolens* by IPCR, forward primers were (5′-AATGTCACGGGTAACTAATTCTA-3′(F-1) and 5′-ATAAAGTAAGAATGTCACGGGTA-3′(F-2), and reverse primers were 5′-GCTTTCACCTTCAACAACAAATG-3′(R-1) and 5′-GTAGATGAGATGATTGGGCGAAAA-3′(R-2). To construct the Psu-miR475b-overexpression vector driven by Psu-miR475b promoter, the amplified *Psu-MIR475b* gene and its promoter were digested with *Xba* I and *Sac* I, and joined by ligation with T4 DNA Ligase, followed by PCR amplification to generate Psu-miR475b-promoter/MIR475b construct (*Pro*_*MIR475b*_*:MIR475b*). For a negative control, empty effector plasmid 35S/Em was constructed by the replacement of GUS gene with a native sequence 5′-TCTAGAGGATCCAATTGCTACCGAGCTC-3′ in pBI121. These constructs were first introduced into *Agrobacterium tumefaciens* strain GV3101 via the freezing-thaw method, and then transferred into *P. suaveolens* by the leaf disc transformation method.

### Promoter-GUS chimeric vector construction and tobacco transformation

A series of 5′ progressive deletions of Psu-miR475b promoter, covering different regions from −939 to −1, −569 to −1, −412 to −1, −351 to −1, −260 to −1, −95 to −1, −50 to −1 and −20 to −1, were respectively generated by PCR using a 5′ sequence of *Psu-MIR475b* as a template with forward primers containing *Hind* III restriction site (underlined): 5′-CCCAAGCTTCTAATAAACTCCATTCTCC-3′(f1), 5′-CCCAAGCTTTTTTCATGTTTGGTGA-3′(f2), 5′-CCCAAGCTTTTTGATTTATGGGTTTTT-3′(f3), 5′-CCCAAGCTTTATTGATTTTGGTTTG-3′(f4), 5′-CCCAAGCTTAAAAAGACACCTGTTT-3′(f5), 5′-CCCAAGCTTGCCTTTTAGGGTTTT-3′(f6), 5′-CCCAAGCTTGAGGGCATCCTTTTCCT-3′(f7), 5′-CCCAAGCTTATACTCAAGATGA-3′(f8), and a common reverse primer containing a *Xba* I restriction site (underlined): 5′-CTCAAGATGAGCAGATTGCTCTAGAGC-3′(r01). Each of the PCR amplified fragments was digested with *Xba* I and *Hind* III (Promega, USA) and purified with TIAN-quick Midi Purification Kit (TIANGEN, Beijing, China). They were then fused to the *GUS* reporter gene of the modified pBI121 vector (Clontech, USA) harboring an *Xba* I site immediately downstream of the *Hind* III site, which was previously digested with *Xba* I and *Hind* III to release 35S promoter. The resulting vectors, confirmed by DNA sequencing, were respectively named as Pro_MIR475b_-939/GUS, Pro_MIR475b_-569/GUS, Pro_MIR475b_-412/GUS, Pro_MIR475b_-351/GUS, Pro_MIR475b_-260/GUS, Pro_MIR475b_-95/GUS, Pro_MIR475b_-50/GUS and Pro_MIR475b_-20/GUS.

The chimeric vectors for tobacco transformation were performed by the same method of *P. suaveolens*. The putative transgenic plantlets resistant to kanamycin were further confirmed by PCR, as well as preliminary GUS staining. The verified transgenic tobaccos were then propagated and synchronized from primary transformants in MS medium. One month-old *in vitro*-grown plantlets were used for subsequent experiments.

### Plant treatment

For inducible expression analysis of GUS activity, the *in vitro* transgenic tobacco plants were sprayed with 200 μM MeJA, 200 μM GA, 5 mM SA, 100 μM ABA, and then incubated at 23 °C for 24 h. To test the effects of the different defense-related stimuli on the activity of Psu-miR475b promoter, the aerial parts between the second and fourth leaves of the *in vitro* transgenic *P. suaveolens* plants were sprayed with 200 μM MeJA, 200 μM GA, 5 mM SA or 100 μM ABA solutions, and the poplar samples were then harvested at time points 0, 6, 12, 24, 36 and 48 h post-treatment. All these treatments were described by our previous studies^56^, and untreated plantlets and plantlets treated with distilled water were used as the controls.

### Histochemical and fluorometric GUS assay

For histochemical staining of GUS, fresh tissue samples were dissected from tobacco plants and immediately subjected to the X-Gluc solution^56^. After overnight incubation at 37 °C, stained samples were bleached with 70% (v/v) ethanol and observed with OLYMPUS BX61 and SZX12 microscopes.

A fluorometric GUS assay was performed as previously described^57^ and the tobacco stem tissues were ground in liquid nitrogen and homogenized in freshly prepared GUS extraction buffer (50 mM NaH_2_PO_4_, pH 7.0, 10 mM EDTA, 0.1% Triton X-100, 0.1% (w/v) sodium laurylsarcosine, 10 mM β-mercaptoethanol). After centrifuging for 15 min at 12,000 rpm at 4 °C, the GUS activity of the supernatant was determined using 4-methylumbelliferyl glucuronide (4-MUG) as a substrate. The fluorescence of the GUS-catalyzed hydrolysis reaction product, 4-methylumbelliferone (4-MU), was measured with the TECAN GENios system. Protein concentration in supernatant was assessed by the Bradford method^58^, using bovine serum albumin as a standard. GUS activity was normalized to the protein concentration of each supernatant extract and calculated as pmol of 4-MU per milligram of soluble protein per minute.

### Gene expression analysis

Genomic DNA was extracted from the mature leaves of *P. suaveolens* with a Plant Genomic DNA Kit (TIANGEN, Beijing, China). Total RNA was isolated from the tested samples using SV Total RNA Isolation System (Promega, Madison, WI, USA), and treated with RNAse-free DNAse I to eliminate the residual genomic DNA, according to the manufacturer’s instructions (Promega, Madison, WI, USA).

Relative quantification of the expressions for Psu-miR475b and its targets, and *GUS* gene by quantitative real-time PCR (qRT-PCR) were performed on 7500 Real-Time PCR System, by using MiRcute miRNA SYBR Green Kit (TianGen, Beijing, China) and SuperScript^TM^ III Platinum^®^ Two-Step qRT-PCR Kit with SYBR^®^ Green (Invitrogen, Carlsbad, CA, USA), respectively. According to the previous reports^59^,^60^ and our generated Solexa sequencing (data not shown), miR167e and miR168a-3p were used as inner references for Psu-miR475b, and poplar *ACTIN* gene as endogenous reference gene for miR475b-targets^27^. Data were from at least three quantitative PCR replicates per sample and three biological replicates. The specific primers of Psu-miR475b and its target genes, *GUS* gene and reference gene are shown in Supplementary Table S2.

### Prediction of *cis*-acting elements within Psu-miR475b promoter

The putative *cis*-elements of Psu-miR475b promoter were identified by online programs including Plant CARE (http://bioinformatics.psb.ugent.be/webtools/plantcare/html/), PLACE (http://www. dna.affrc.go.jp/ PLACE/ signalup.html), TESS (http://www.cbil.upenn.edu/cgi-bin/tess/tess) and PWM (http://users.soe.ucsc.edu/~kent/improbizer/motifMatcher.html).

## Additional Information

**How to cite this article**: Niu, J. *et al.* Cross-talk between freezing response and signaling for regulatory transcriptions of *MIR475b* and its targets by miR475b promoter in *Populus suaveolens. Sci. Rep.* **6**, 20648; doi: 10.1038/srep20648 (2016).

## Supplementary Material

Supplementary Information
