# Supplementary material for: Cross-talk between freezing response and signaling for regulatory transcriptions of MIR475b and its targets by miR475b promoter in Populus suaveolens
Source: Sci Rep. 2016 Feb 8;6:20648. doi: 10.1038/srep20648 (PMC4745078; doi:10.1038/srep20648)
Supplement: Supplementary Information [file srep20648-s1.doc]

**Cross-talk between freezing response and signaling for regulatory transcriptions of *MIR475b* and its targets by miR475b promoter in *Populus suaveolens***

**Jun Niu^a^, Jia Wang^a^, Huiwen Hu, Yinlei Chen, Jiyong An, Jian Cai, Runze Sun, Zhongting Sheng, Xieping Liu, Shanzhi Lin^*^**

^1^College of Biological Sciences and Biotechnology, National Engineering Laboratory for Tree Breeding, Key Laboratory of Genetics and Breeding in Forest Trees and Ornamental Plants, Ministry of Education, Beijing Forestry University, Beijing 10083, China

^a^These authors contributed equally to this work

**^*^**Corresponding author: Tel/Fax +86-10-62336114; szlin@bjfu.edu.cn


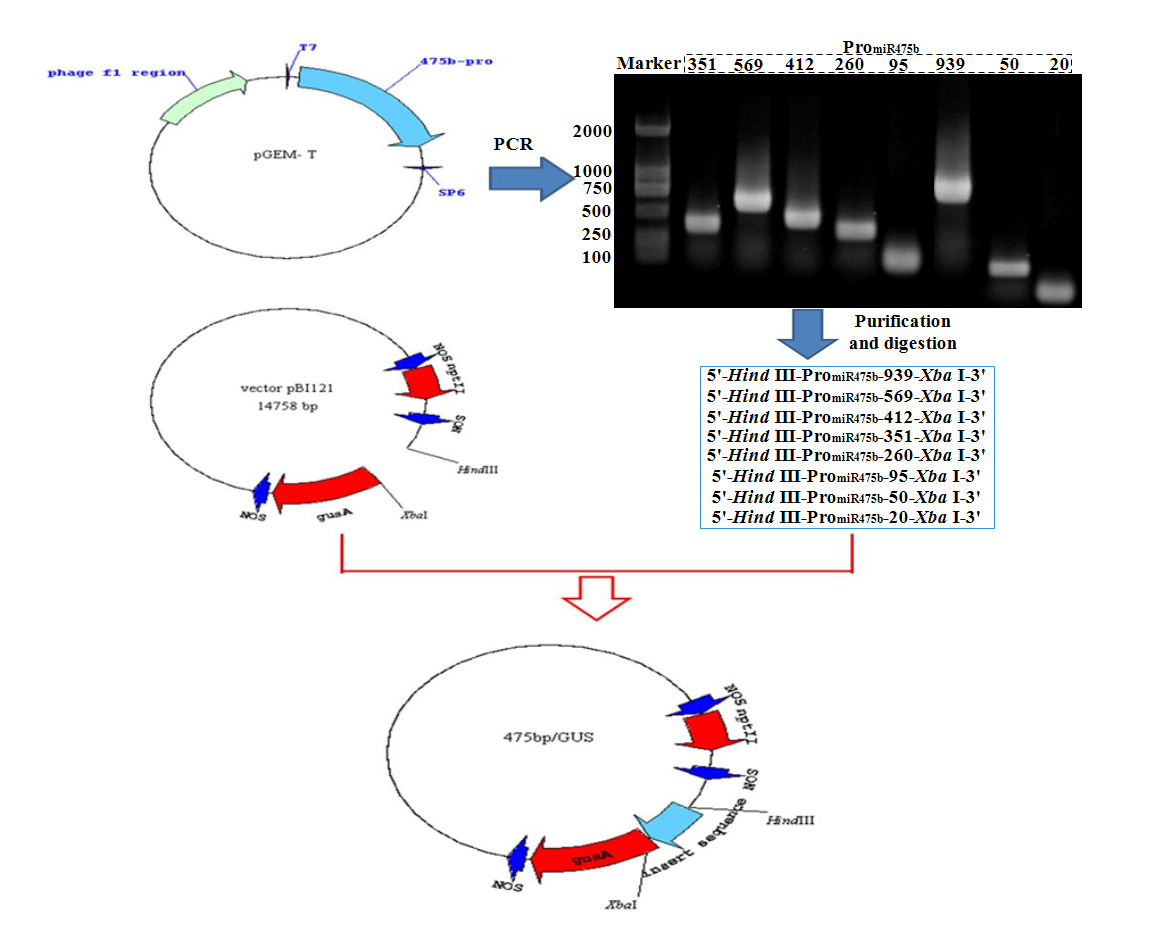


**FigureS1. Construction and identification of different Pro_MIR475b_-GUS chimeric expression vectors.**

A series of 5′ promoter deletion, covering different regions from -939 to -1, -569 to -1, -412 to -1, -351 to -1, -260 to -1, -95 to -1, -50 to -1 and -20 to -1, replaced the CaMV 35S promoter and then were inserted into pBI121 vectors used for transgenic tobacco.


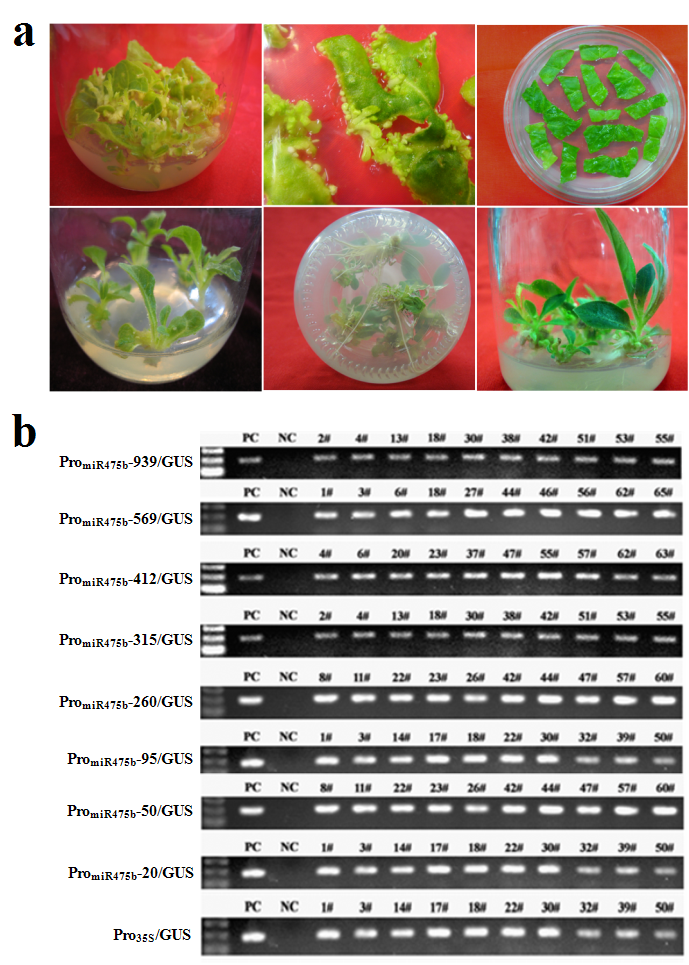


**Figure S2. Generation and identification of transgenic** *Pro_MIR475b_:GUS* and *Pro_35S_:GUS* **tobacco plants. (a)** Generation of putative transgenic *Pro_MIR475b_:GUS* and *Pro_35S_:GUS* tobacco plants. **(b)** Identification of transgenic tobacco plants by PCR. PC, positive control (the respective transformed vector); NC, negative control (untransformed tobacco).


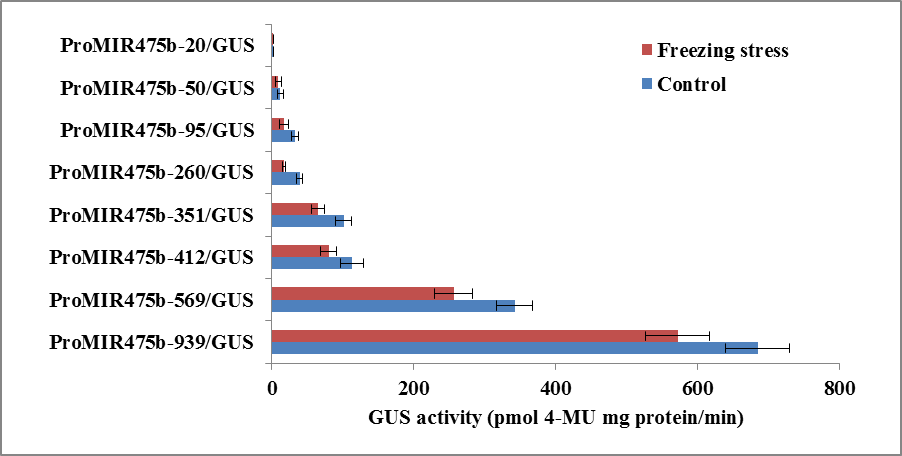


**Figure S3.** The fluorometric GUS assay for a series of 5′ deletions of Psu-miR475b promoter in transgenic tobacco stem under freezing stress.


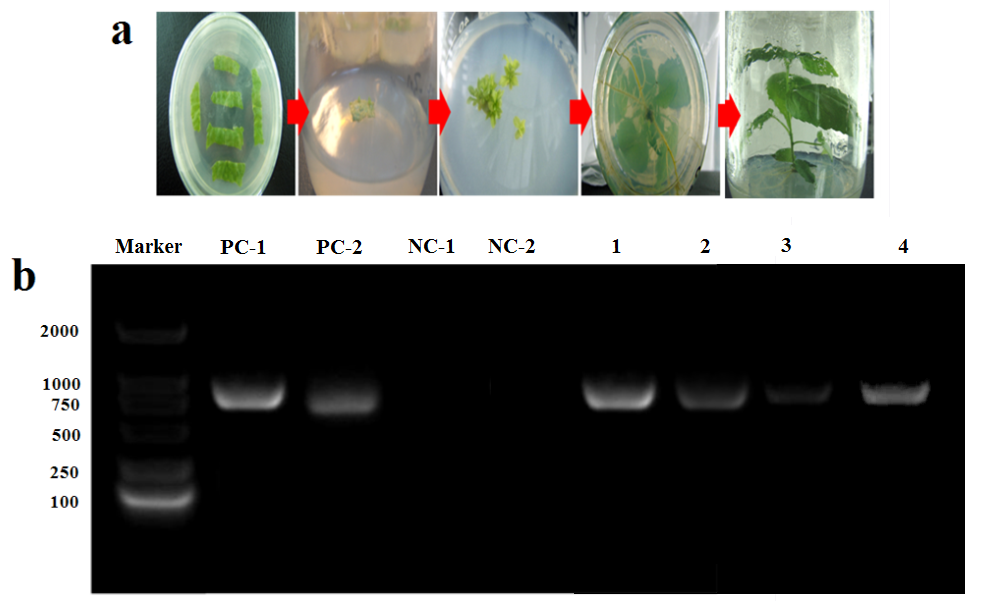


**Figure S4. Generation and identification of transgenic *Pro_MIR475b_:MIR475b* and *Pro_35S_:MIR475b P. suaveolens*. (a)** Generation of putative transgenic *Pro_MIR475b_:MIR475b* and *Pro_35S_:MIR475b P. suaveolens* plants. **(b)** Identification of transgenic *P. suaveolens* plants by PCR. PC-1 and PC-2 respectively represented transformed vectors used as positive controls, NC represented untransformed tobacco used as negative control, 1 and 2 were transgenic *Pro_MIR475b_:MIR475b* lines, and 3 and 4 were transgenic *Pro_35S_:MIR475b* lines.

**Supplementary Table S1 C*is*-acting elements in the *Psu-MIR475b* gene promoter**

| Putative *cis*-elements | Sequence | Position/bp | Function |
| --- | --- | --- | --- |
| 5' UTR Py-rich stretch | TTTCTTTTCT | -838/-829 | conferring high transcription level |
| AACA motif | TAATAAACTCCA | -938/-929 | involved in endosperm-specific negative expression |
| ARE | TGGTTT | -342/-339 | essential for the anaerobic induction |
| CAAT box | CAAT | -149/-146, -591/-588  -666/-663, -757/-754  -786/-783 | common *cis*-acting element in promoter and enhancer regions |
| TC-rich repeats | ATTTTCTCCA | -687/-678 | involved in defense and stress responsiveness |
| GARE motif | AAACAGA | -452/-446 | involved in gibberellin responsiveness |
| TCA element | AAGAAAAGGA | -297/-288 | involved in salicylic acid responsiveness |
| CGTCA motif | CGTCA | -76/-71 | involved in methyl jasmonate responsiveness |
| TATA box | TTTAAAAA | -32/-25 | core promoter element for transcription start |
| CG motif | CCATGGGG | -57/-50 | involved in light responsiveness |
| Box 4 | ATTAAT | -360/-355, -620/-615 |
| GT1 motif | GGTTAAT | -550/-544 |
| I-box | GATATGG | -635/-629 |
| GAG motif | AGAGATG | -817/-811 |

***Supplementary Table S2 Specific primers used for 3'/5'-RACE and qRT-PCR of Psu-miR475b and its target genes and GUS report gene***

| Gene | Forward primer | Reverse primer |
| --- | --- | --- |
| miR475a 3' GSP1 | CCAAGGTTGCGCTTCTGGCTTTCA | - |
| miR475a 3' GSP2 | TTGCTGAAATGTGCGACCAAATAG | - |
| miR475b 5' GSP1 | - | ATAACATCTTAATCAATGGGCACT |
| miR475b 5' GSP2 | - | GATCAAGATGTTATGACTATGAAAGC |
| XM_002319013.1 | TACAGTGCTTTGATGGACGGATAC | TCGGGAGTCAAATCTCTGTCATAC |
| XM_002325743.1 | CTACGATGCTGTGATTTCCCTT | CAGGTTGACACCCTCTTGCTAC |
| XM_002336177.1 | CAACGTGTTGATCTTGGGTTC | AGCCTCATTCACCCGCCTATCTT |
| XM_002329199.1 | GACGATGCCCTTGCTTCTTTCA | GAGTTGTAACCTCTTTCCACCG |
| DB891597.1 | ACCCACTATTGTCACATTTACC | ATCCAGCAGCCGCAGCAGTTTC |
| XM_002309526.1 | GGTCTTCAACCCGATGCTGT | GACAGTAGGTTGACACCCGCTTG |
| XM_002301639.1 | TGGGCAGCAAAGGTTACTCT | TGTAAGAGTGAAATACCAGGAG |
| XM_006377350.1 | CCTTCCTTACAGTGCCCAATGAT | GCTTCCTTCTTTACAGAGCCCATT |
| XM_002310640.2 | GCATCAATGGGTACTGCAAGGT | CGCATCCCGAGGTCTCAAAG |
| XM_006389244.1 | CCTGTAAGAGTGGAGGGATTGATG | CAATTCGGAAGCAGGCCATA |
| XM_006389560.1 | GTGTATGTACGTGGCTTGGTTCC | CGCACTTTCAATGTTCCCTTCC |
| Psu-miR475 | CGCTTACAGTGCCCATTGATTAAGA | - |
| Tobacco *ACTIN* | CTGCTGGAATTCACGAAACA | GCCACCACCTTGATCTTCAT |
| Poplar *ACTIN* | ACCCTCCAATCCAGACACTG | TTGCTGACCGTATGAGCAAG |
| miR167e | GAAGCTGCCAGCATGATGTG | - |
| miR168a-3p | CCCGCCTTGCATCAACTG | - |
